# Familial acute aortic dissection associated with a novel *ACTA2* germline variant

**DOI:** 10.1007/s00428-022-03366-9

**Published:** 2022-07-28

**Authors:** Thomas Strecker, Felix Wiesmueller, Sabine Rudnik-Schöneborn, Juliane Hoyer, André Reis, Michael Weyand, Abbas Agaimy

**Affiliations:** 1 Center of Cardiac Surgery, Friedrich-Alexander-University Erlangen-Nuremberg, Östliche Stadtmauerstraße 27, 91054 Erlangen, Germany; 2 Division of Cardiothoracic Surgery, Brigham and Woman’s Hospital, Harvard Medical School, Boston, MA USA; 3 Institute of Human Genetics, University of Innsbruck, Innsbruck, Austria; 4 Institute of Genetics, Friedrich-Alexander-University Erlangen-Nuremberg, Erlangen, Germany; 5 Institute of Pathology, Friedrich-Alexander-University Erlangen-Nuremberg, Erlangen, Germany

**Keywords:** Aortic dissection, ACTA2 mutation, Cardiac surgery, Pathology, Echocardiography, Computer tomography, Human genetics

## Abstract

Aortic dissection is a life-threatening cardiovascular disease. Hereditary disorders are responsible for a small percentage of cases. Nonetheless, it is important to identify genetic causes, as they are often autosomal dominantly inherited and are of life-saving importance if we can identify persons at risk. Mutations of the *ACTA2* gene are the most common cause of non-syndromic familial aortic disease. Exploration of the genetic background in suspected familial cases and determination of the exact etiology are mandatory for management and establishing appropriate follow-up strategies due to the risk of fatal recurrences. Herein, we present a 21-year-old male with a familial acute aortic dissection associated with novel *ACTA2* germline variant and discuss the management and surveillance considerations.

## Introduction

Aortic dissection is a very serious and often life-threatening disease, mainly caused by degenerative disorders of the connective tissue of the aortic wall. Familial aortic dissection is rare and needs prompt clinical management and monitoring. Atherosclerotic disease and arterial hypertension are among the major causes of aortic dissection. Uncommon conditions predisposing to this disease include disorders associated with weakness and/or degeneration of the aortic wall connective tissue, in particular Marfan syndrome, idiopathic media degeneration (Erdheim-Gsell), and other rare conditions. Deleterious mutations in genes encoding different connective tissue elements cause a diversity of diffuse vasculopathies such as thoracic aortic aneurysms and dissections as well as occlusive vascular diseases [1]. Germline mutations of the *ACTA2* gene are responsible for 12–21% of familial non-syndromic thoracic aortic disease [2]. Main clinical manifestations are Stanford type A aortic dissection (54% at a median age of 36 years) and type B dissection (21% at a median age of 27 years). Aortic events are more prevalent in males (62%) than in females (38%). Other clinical features—mostly restricted to specific mutations—include occlusive vascular disease (premature coronary artery disease, Moya-Moya like disease), congenital heart disease, and iris flocculi [3]. It is highly recommended to adopt an etiology-based long-term monitoring for aortic aneurysms in high-risk patients [4]. Transesophageal echocardiography (2D-transthoracic echocardiography; 2D-TTE) and computer tomography are the standard diagnostic tools to clarify acute pathology of the ascending aorta [5].

## Clinical summary

A 21-year-old Caucasian male was referred to the emergency department of our hospital soon after he collapsed at home in the early morning hours. He noted progressive chest pain associated with dyspnea and felt a stabbing pain between the shoulders with subsequent dizziness, fatigue, and arterial hypotension. The patient’s family history included several family members with acute aortic dissection. His mother had undergone replacement of the thoracic aorta at the age of 36, approximately 4 years before we performed emergency surgery on the patient described. The grandfather on the mother’s side had died of aortic dissection at the age of 56 years. Furthermore, his great-grandmother on the grandfather side and one of her brothers also had succumbed to the very same condition. Familial clustering of the disease suggested an autosomal dominant condition. Genetic testing was initially offered to the patient’s mother (index patient). A genetic variant in the *ACTA2* gene was detected in the patient’s mother which was not known in the genetic databases and therefore initially classified as variant of unknown significance.

**Fig. 1:**
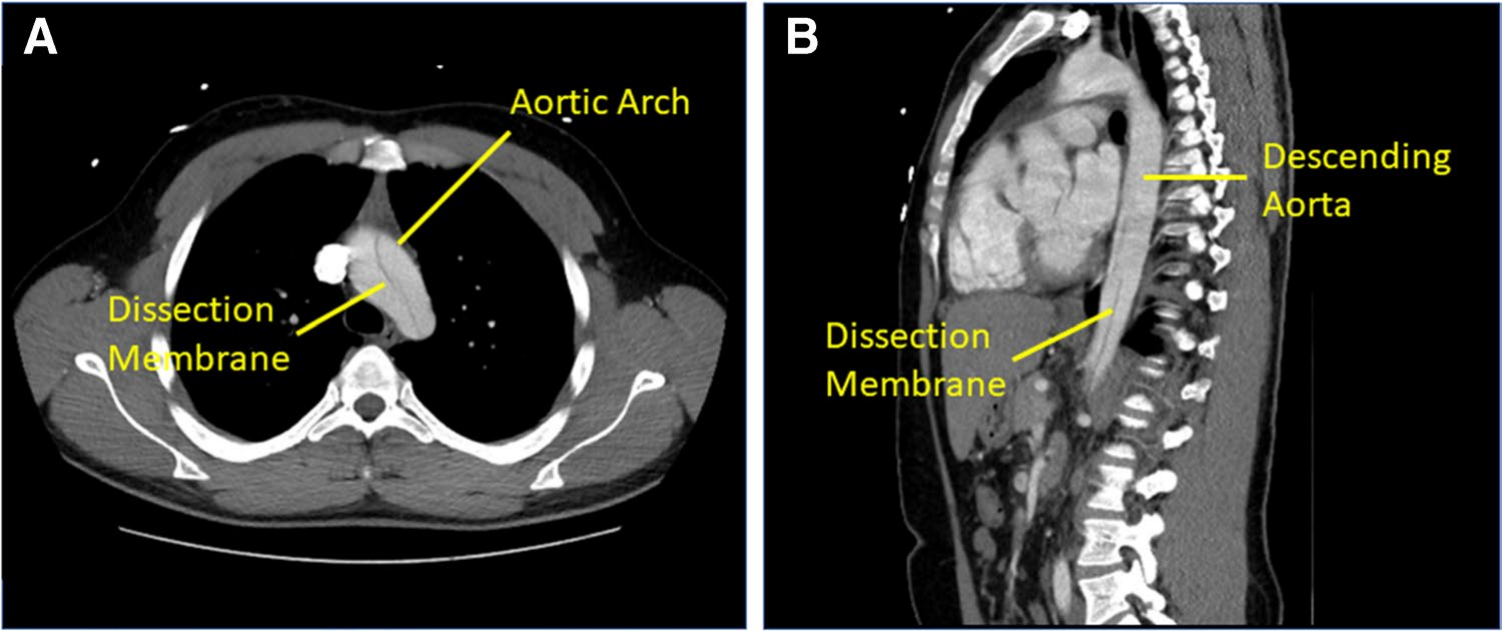
**A** Computed tomography (CT) demonstrated the aortic dissection and the aneurysm of the ascending aorta and the aortic arch. **B** The aortic dissection reached down the descending aorta to the level of the mesenteric artery

Upon admission to the hospital, the patient’s blood pressure was 120/60 mmHg and his heart rate was 99 beats per minute (bpm). Transthoracic echocardiography (TTE) and abdominal sonography depicted ascending aortic, aortic arch, and descending aortic aneurysms with ambiguous floating dissection. The aortic annulus was dilated; the aortic valve was tricuspid and insufficient. Subsequently, computed tomography (CT) confirmed aortic dissection starting in the ascending aorta down to the descending aorta to the level of the mesenteric artery (Fig. 1A, B).

The patient was transferred to the operating theater urgently, where a median sternotomy was performed and a cardiopulmonary bypass was installed through the brachiocephalic trunk and right atrium cannulation. Intraoperatively, there was a small amount of hemopericardium and the ascending aorta appeared dilated and partially hemorrhagic. The aneurysm of the ascending aorta extended to the brachiocephalic trunk origin. Longitudinal aortotomy demonstrated the circular dissection starting approximately 15 mm above the aortic annulus, leaving the coronary arteries and ostia unaffected. The aortic annulus was slightly dehiscent. It was resutured in the anatomic position with a Dacron 26-mm aortic prosthesis replacing the ascending aorta. Subsequently, the patient was weaned from cardiopulmonary bypass without any signs of cardiac failure.

The further postoperative recovery was complicated due to a progressive multiple organ dysfunction. With increasing deterioration, a new CT scan of the thorax and abdomen was performed on the first postoperative day. The examination showed a reduced perfusion of the celiac trunk, the mesenteric artery, and the right kidney. Afterwards, a decompression through fenestration between the right and false lumen of the descending aorta was performed. However, serum lactate and transaminases were significantly elevated the day after. CT scan of the abdomen revealed that the true lumen of abdominal aorta had collapsed despite prior efforts. Consequently, a decision by interdisciplinary consensus for emergency procedure was made. Incision of the left parietal peritoneum with mobilization and medial reflection of stomach, tail of pancreas, left kidney, and spleen allowed for immediate access to the abdominal aorta (Mattox maneuver) [6]. Then, the aortic dissection plane was resected and stabilized by suturing the aortic intima to the foot of the celiac artery branch. An intraoperative ultrasound showed sufficient flow through aorta and celiac artery. Sufficient perfusion of abdominal organs was confirmed by postoperative CT scan. Due to the reduced perfusion of the kidneys, the patient underwent continuous hemofiltration for 8 days followed by intermittent dialysis. After recovery, the patient was finally discharged to the hospital near his hometown and later home.

### Pathological findings

The resected specimen was taken from the ascending aorta. The histopathological findings were described in accordance with the recently published consensus paper of the Society for Cardiovascular Pathology and the Association for European Cardiovascular Pathology [7].

**Fig. 2:**
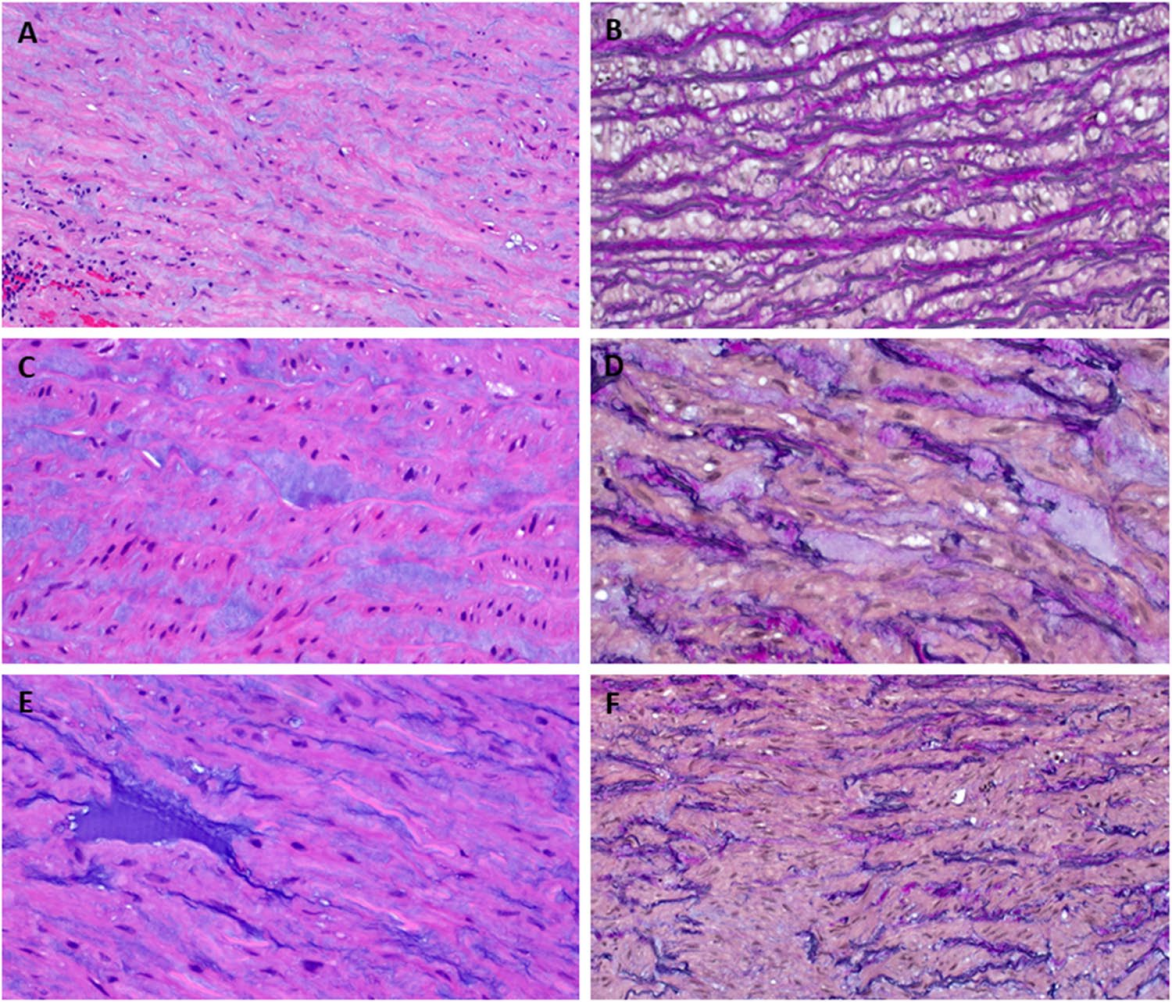
Representative examples of the histopathological changes in the thoracic aorta. **A** Diffuse intra-lamellar accumulation of extracellular mucoid matrix (H&E stain, ×100). **B** Elastica van Gieson (EvG) stain highlighting mild thinning and separation of elastic fibers. This finding, which is close to the normal pattern, is in line with the significant heterogeneity of the aortic wall damage characteristic of this disease (EvG stain, ×100). **C** More advanced/severe but still mainly intra-lamellar accumulation of extracellular mucoid matrix (H&E stain, ×200). **D** EvG stain of same field highlighting severe loss, separation, thinning, and fragmentation of the elastic fibers (EvG stain, ×200). **E** Both diffuse intra-lamellar and translamellar accumulation of extracellular mucoid matrix (H&E stain, ×200). **F** EvG highlighting the interruption of elastic fibers (EvG stain, ×100)

**Fig. 3:**
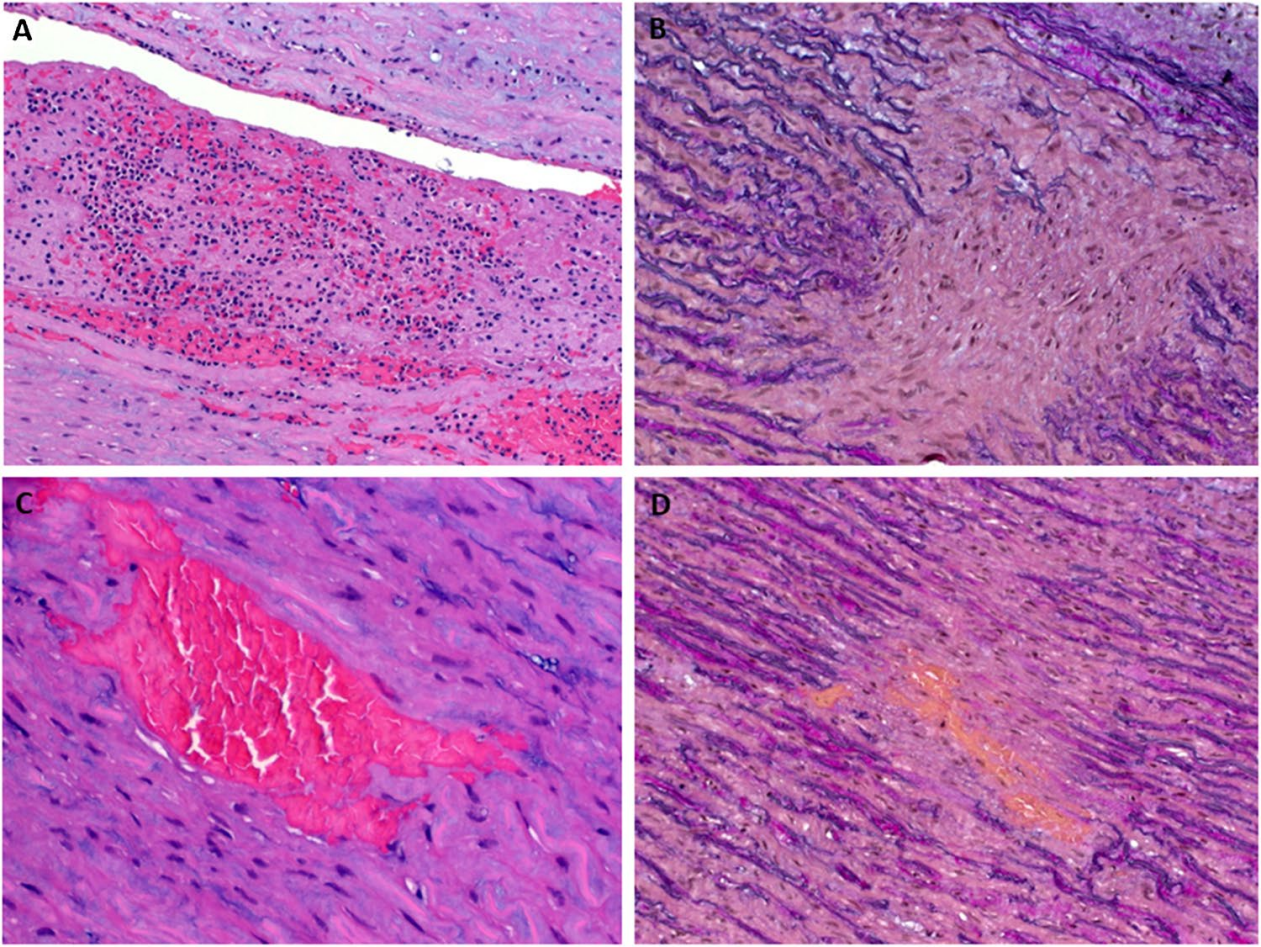
**A** Section of the aortic wall showing the presence of blood/thrombus within the dissection (false lumen) (H&E stain, ×100). **B** Sections from grossly unremarkable aortic wall showed the interruption of the elastic fibers by multifocal old fibrous scars (EvG stain, ×200). **C** Focus of microhemorrhage within grossly normal-looking aortic wall, consistent with focal acute dissection (intramural hematoma) (H&E stain, ×200). **D** EvG highlighting the fibrosis surrounding ectatic vessels and hemorrhagic foci (EvG stain, ×200)

The preparation revealed extensive dissection separating the thoracic aortic intima at variable thickness from the deeper aortic wall. The dissection plane (false lumen) was filled with fibrin-containing bloody material. The tunica media revealed moderate to severe degree of medial degeneration around and away from the dissection plane highlighted by severe multifocal/diffuse intra-lamellar accumulation of extracellular mucoid matrix associated with extensive moderate translamellar accumulation, moderate extensive elastic fiber fragmentation and loss, and patchy extensive smooth muscle cell loss (summarized in Table 1 and illustrated in Figs. 2 and 3). Multiple foci of scarring indicating healed microruptures were seen in the aortic wall. There was no evidence of aortitis of any type, granulomatous or giant cell reaction.

**Table 1:** Summary of the histopathological findings in the thoracic aortic specimen

Feature	Severity/extent
Intra-lamellar MEMA	Severe multifocal/extensive
Translamellar MEMA	Moderate extensive
Elastic fiber fragmentation and/or loss	Moderate extensive
Smooth muscle cell nuclei loss	Patchy extensive
Laminar medial collapse	Absent
Overall medial degeneration	Moderate to severe

*MEMA*, mucoid extracellular matrix accumulation

### Methods of genetic testing

Peripheral blood sample from the mother: massive parallel sequencing: enrichment of the genes of interest with Nextera® Rapid Capture (TruSightTM One Panel, Illumina) from genomic DNA and massive parallel sequencing (NextSeq, Illumina). Automated alignment to the sequence of the human reference sequence GRCh37 (hg19), data analysis with the SeqNext® (JSI) software (including copy number variation), additionally with CNV Detective software (Institute for Bioinformatics, JKU Linz).

Analysis criteria: evaluation of genes responsible for thoracic aortic aneurysm disease (TAAD): *ACTA2*, *MYH11*, *MYLK*, *TGFBR2*, *SMAD3*, *TGFBR1*, *TGFB2*, *TGFB3*, *FBN1*, *COL3A1*, *NOTCH1*. A minimum of 20× coverage is applied for the coding and the adjacent intronic sequences (−15/+5). Disease relevant mutations (classes 4 and 5 of the 5-class system) by [8] variants with uncertain significance (VUS, class 3) are reported. The classification of mutations follows the consensus recommendations of the American College of Medical Genetics [9]. The A in the start codon corresponds to c.1 [10].

Filter criteria: (1) MAF <1%, (2) mutation frequency in reads >20%, (3) stop-, missense-, and frameshift-mutations, as well as mutations in the canonical splice site.

Peripheral blood sample from the index patient: exome sequencing was performed on an Illumina HiSeq system (Illumina, Inc., San Diego, CA), after enrichment with Twist Human Comprehensive Exome Enrichment Technology (TWIST Bioscience). Automated alignment to the human reference sequence (hg19) with the Burrows-Wheeler-Aligner. Copy number analysis (deletions/duplications) was performed with ExomeDepth Software. Variant data were analyzed with our custom NGS Variant Analyzer tool, which involves the semiautomatic selection and data quality inspection of variants.

Analysis criteria: evaluation of 102 genes responsible for thoracic aortic aneurysm disease (TAAD) or connective tissue/vessel disease: ABCC6, ADAMTS2, ADAMTSL2, ADAMTSL4, AEBP1, ALDH18A1, ALPL, ANO5, ATP6V0A2, ATP6V1A, ATP6V1E1, ATP7A, B3GALT6, B4GALT7, BMP1, BGN, C1R, C1S, CBS, CHST14, COL11A1, COL11A2, COL12A1, COL1A1, COL1A2, COL2A1, COL3A1, COL4A1, COL4A2, COL5A1, COL5A2, COL6A1, COL6A2, COL6A3, COL9A1, COL9A2, CREB3L1, CRTAP, DSE, DSPP, EFEMP2, ELN, EMILIN1, FBLN5, FBN1, FBN2, FGFR2, FKBP10, FKBP14, FLNA, GORAB, FOXE3, GATA5, IFITM5, LOX, LRP5, LTBP2, LTBP3, LTBP4, MAT2A, MED12, MFAP5, MYH11, MYLK, NOTCH1, P3H1, PHYKPL, PLOD1, PLOD2, PLOD3, PLS3, PPIB, PRDM5, PRKG1, PYCR1, RIN2, ROBO4, SEC24D, SERPINF1, SERPINH1, SKI, SLC2A10, SLC26A2, SLC39A13, SMAD3, SMAD4, SMAD6, SOX9, SP7, SPARC, STAT3, TGFB1, TGFB2, TGFB3, TGFBR1, TGFBR2, TMEM38B, TNXB, UPF3B, WNT1, ZDHHC9, ZNF469. An average of >100× coverage is applied for the coding and the adjacent intronic sequences. Disease relevant mutations (classes 4 and 5 of the 5-class system) and variants with uncertain significance (VUS, class 3) are reported. The classification of mutations follows the consensus recommendations of the American College of Medical Genetics [9].

Filter criteria: (1) variant frequency in normal population of <21%, (2) at least 3 novel alleles, (3) synonymous and non-synonymous variants in the coding region and in the canonical splice site.

### Results of genetic testing

Genetic analysis of the patient’s mother peripheral blood sample revealed a missense mutation c.283G>A, p. (Glu95Lys) in the *ACTA2* gene which has been classified as a variant of uncertain significance (VUS, class 3). It is reported only once without further clinical details in the ClinVar database (Oct. 2019). This variant was not observed in large population cohorts (ExAC, ESP, 1000 Genomes Consortium). The Glu95Lys amino acid substitution is non-conservative, which is likely to impact secondary protein structure, and it occurs at a position that is conserved across species. In silico analysis predicts that this variant is probably damaging to the protein structure or function (SIFT v.6.2.0: deleterious, score: 0, median 4.32; Mutation Taster v2013: disease causing, *p* value: 1).

**Fig. 4:**
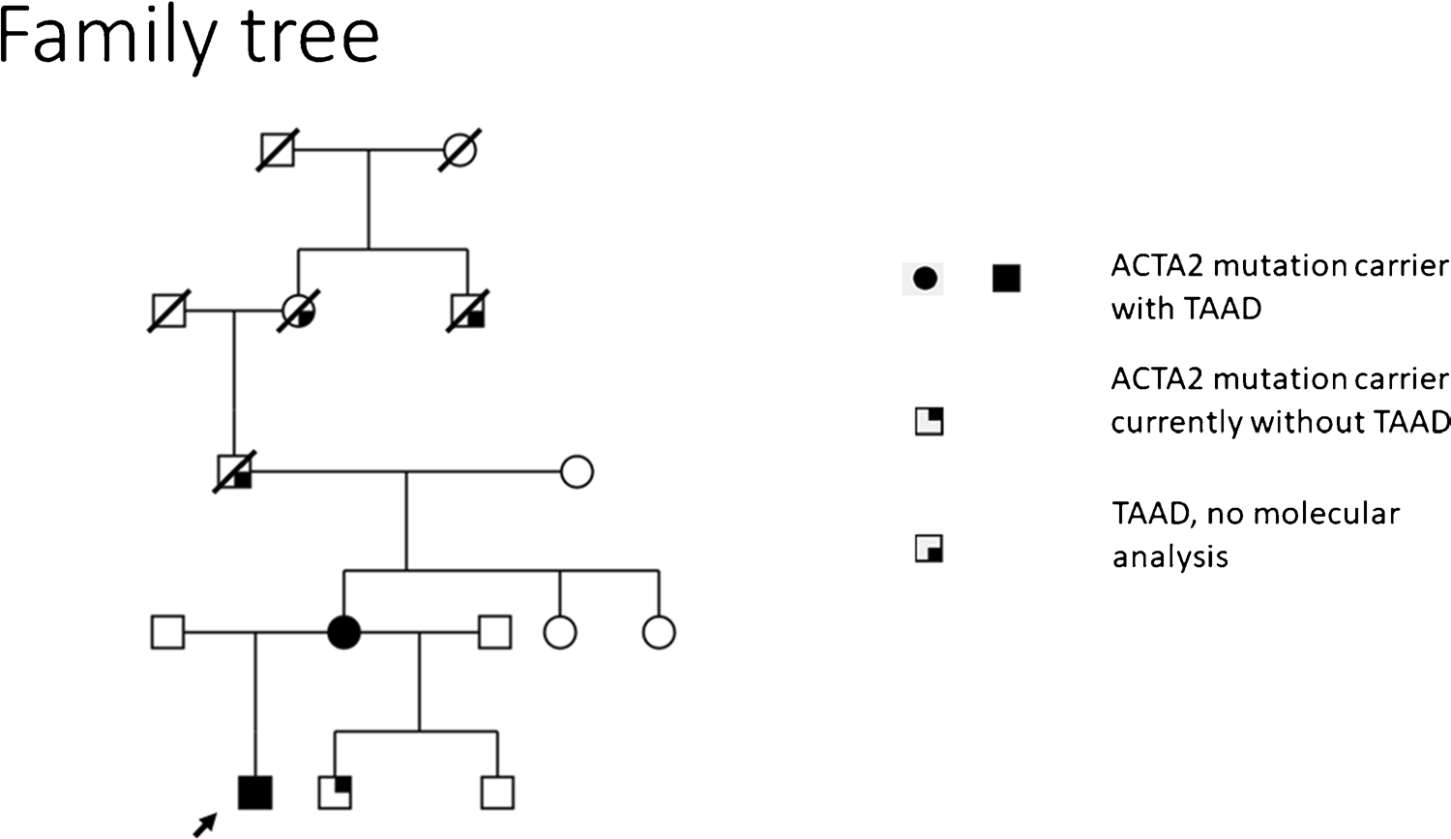
Complete genetic family tree. The arrow marks our patient

Genetic testing of the patient and his siblings revealed that he is also carrier of the same *ACTA2* gene mutation and one of his two younger brothers as well (Fig. 4).

In the recent echocardiography of the 14-year-old brother, who also carries the *ACTA2* gene mutation, the aortic dimensions were found to be in the upper normal range. We suggest regular echocardiographic controls regarding the borderline dilated aortic dimensions. Echocardiography of the 12-year-old youngest member of the family without a gene mutation showed aortic dimensions consistent with age. The heart was structurally and functionally normal.

Recommendations for physical activity or exercise in patients with aortic aneurysms or after surgery for aortic dissection refer primarily to aerobic endurance training. Isometric strength training is not recommended due to the high increase in systolic blood pressure. These are always individual recommendations and they depend on the course of the diameter of the aorta and the underlying disease [11].

## Discussion

Although aortic dissection has been attributed to diverse pathological conditions and acquired etiologies, hereditary factors likely play an important etiological role in the development of such dissections [12]. Up to 20% of affected individual who do not have a known syndrome (as Marfan syndrome) have a family history of thoracic aortic aneurysm and dissection (TAAD).

Non-syndromic familial TAAD is a rare condition that remained of unknown etiology for decades. However, recent studies have uncovered several different gene mutations that are associated with familial TAAD. To date, 16 predisposing genes are known. *ACTA2* missense mutations are the major genetic cause of familial TAAD identified to date and are responsible for disease in 12–16% of familial TAAD cases [1, 13]. This gene which is mapped to chromosomal region 10q23.31 encodes for one of the six major actin isoforms, the α-smooth muscle actin. On a subcellular level, α-smooth muscle actin is an integral part of sarcomeres within smooth muscle cells [14]. If structurally altered, smooth muscle cells cannot contract properly. Arteries, which contain layers of smooth muscles, then are susceptible to media degeneration, overstretching, and tearing. The aorta is the segment of the vascular system that must withstand highest pressures. Hence, any degenerative or other pathological alterations would result in repeated tearing of the elastic and smooth muscle fibers, ultimately leading to formation of aneurysms or dissection at this particular anatomic location.

Thoracic aortic aneurysms in *ACTA2* carriers are typically fusiform and initially involve the aortic root, extending into the ascending aorta and aortic arch. Descending and abdominal aortic aneurysms are less common. A subset of *ACTA2* pathogenic variants predispose to early-onset stroke or coronary artery disease.

The current case represents a family with a presumed high penetrant *ACTA2* gene mutation in the pathogenesis of TAAD. *ACTA2* mutations are inherited in an autosomal dominant manner. In other families, *ACTA2* mutations exhibited reduced penetrance and variable expressivity [1]. The lifetime risk for an aortic event (defined as presentation with an acute aortic dissection or surgical repair of a thoracic aortic aneurysm) is 76%, suggesting that additional environmental or genetic factors play a role in expression of aortic disease in individuals with *ACTA2* mutations [15]. Therefore, predicting the clinical course in individuals of affected families is difficult. Following the 2019 VASCERN consensus statement, cardiological screening should include yearly 2D-TTE along with ophthalmologic examinations in all individuals with a pathogenic *ACTA2* variant, and complete vascular imaging of thorax and abdomen every 2–5 years in persons over 18 years. If an aortic root aneurysm is present, prophylactic surgery is recommended at a diameter of 45–50 mm [3].

## Conclusion

While timely and correct diagnosis of acute aortic dissection is of utmost importance for saving individual patient’s life, recognition of a possible familial background is mandatory for establishing appropriate follow-up strategies and for considering potential prophylactic measures for at-risk individuals.

## Declarations

We have also included the patient’s informed consent in our manuscript. This manuscript was written in accordance with Declaration of Helsinki. Because this manuscript is only a retrospective description of a rare disease, an ethics committee was not necessary. Of course, we also obtained informed consent from the patient to publish the data.
